# Glypican 4 may be involved in the adipose tissue redistribution in high-fat feeding C57BL/6J mice with peroxisome proliferators-activated receptor γ agonist rosiglitazone treatment

**DOI:** 10.3892/etm.2014.1998

**Published:** 2014-09-30

**Authors:** LI LIU, HAILUN GU, YUE ZHAO, LI AN, JUN YANG

**Affiliations:** 1 Department of Nutrition and Food Hygiene, School of Public Health, China Medical University, Shenyang, Liaoning 110001, P.R. China; 2 Department of Orthopedics, Shengjing Hospital, China Medical University, Shenyang, Liaoning 110004, P.R. China

**Keywords:** glypican 4, obesity, subcutaneous fat, visceral fat, peroxisome proliferators-activated receptor γ

## Abstract

Fat distribution affects the risk of developing obesity-related chronic diseases. Glypican 4 (Gpc4) may be involved in the regulation of obesity and body fat distribution. The aim of the study was to explore whether Gpc4 affects fat accumulation and the possible mechanism. C57BL/6J mice were fed with a high-fat diet for eight weeks and treated with a peroxisome proliferators-activated receptor γ (PPARγ) agonist, rosiglitazone, for another four weeks. The weight of inguinal and epididymal fat pads was determined. The *Gpc4* mRNA and protein expression and two probable regulators of the *Gpc4* gene, specificity protein 1 (*Sp1*) and *Sp3* mRNA, were also measured. Mice treated with rosiglitazone showed a significant increase in subcutaneous fat weight compared with the untreated mice. The expression of *Gpc4* mRNA and protein was significantly higher in visceral than in subcutaneous fat in all the groups. Compared with untreated mice the expression of *Gpc4* and *Sp3* mRNA in subcutaneous fat and the expression of *Sp1* and *Sp3* mRNA in visceral fat in mice treated with rosiglitazone increased significantly. The *Sp3*/*Sp1* ratio was consistent with the expression of *Gpc4* mRNA and protein in subcutaneous and visceral fat. The present study indicated that *Gpc4* may play an important role in fat distribution, and this effect is perhaps regulated by the ratio of Sp3/Sp1 in the subcutaneous and visceral fat tissues.

## Introduction

Obesity has become a severe public health problem worldwide and it is closely associated with specific chronic diseases, including type 2 diabetes, metabolic syndrome and certain cancers. However, the risk of developing these disorders is different in two obesity types. Fat distribution in subcutaneous and visceral adipose tissues have different influences on developing these disorders (1). Individuals who exhibit peripheral obesity (fat mainly accumulated in the gluteofemoral region) are at little or no risk of obesity-related diseases, whereas individuals who exhibit central obesity (fat mainly distributed in visceral depots) are prone to developing these complications. However, depot-specific regulation of adipocyte genes and the mechanisms underlying depot-differences in the metabolism and function of subcutaneous and visceral fat depots thus far remain to be investigated.

Glypican 4 (Gpc4), a member of the heparan sulfate proteoglycan family, plays an important role in the regulation of cell growth, differentiation and morphogenesis (2). Gpc4 also acted as an essential modulator of key regulatory proteins, including Wnt, bone morphogenetic proteins, fibroblast growth factor and sonic hedgehog (3). The expression level of Gpc4 may contribute to the regulation of preadipocyte differentiation by means of regulating the binding of growth and differentiation factors to their cognate high affinity, signal-transducing receptors (4). The expression level of *Gpc4* mRNA presents a clear difference in subcutaneous and visceral adipose tissues. There were strong correlations of *Gpc4* expression with body mass index (BMI) and waist/hip ratio (WHR) in human adipose, which indicated that *Gpc4* may play an important role in obesity and body fat distribution (5). Our recent study showed that the filial generation mice had higher epididymal adipose tissue weight and *Gpc4* mRNA expression when the pregnant mice were exposed to low-dose di-2-ethylhexylphthalate (6), indicating that the *Gpc4* gene may be involved in fat accumulation. However, the mechanism of how Gpc4 regulates fat distribution is still not understood.

Peroxisome proliferators-activated receptor γ (PPARγ), a major regulator of adipocyte differentiation, exerts an important role in fat accumulation. A study showed that the PPARγ agonist *in vivo* induces adipose tissue redistribution from visceral to subcutaneous fat (7). The mechanisms that have been proposed include the variation of the differentiation of preadipocytes from subcutaneous and visceral regions (8) and the depot-specific regulation of lipid uptake, storage and energy expenditure genes in visceral and subcutaneous fat (9–11). Another hypothesized mechanism is that PPARγ activation may affect the expression of the *Gpc4* gene in subcutaneous and visceral adipose tissues that are involved in the regulation of fat distribution. Therefore, to verify the hypothesis, in the present study high-fat feeding (HF) C57BL/6J mice were treated with a PPARγ agonist rosiglitazone (RSG) and the effects of PPARγ activation *in vivo* on HF mice and the expression of *Gpc4* mRNA and protein in epididymal and inguinal depots, which were used as representative of visceral and subcutaneous fat respectively, were assessed. The mice were also evaluated *in vivo* for two probable regulators of the *Gpc4* gene, specificity protein 1 (*Sp1*) and *Sp3* to explore the hypothesis that they, perhaps, to a certain degree, are involved in fat distribution regulation of *Gpc4*.

## Materials and methods

### Animals and treatment

Twenty-one male C57BL/6J mice (weight, 18–22 g) were purchased from Shanghai SLAC Laboratory Animal Co., Ltd. (Shanghai, China). The animals were divided into three groups. Mice in the control (CON) group (n=7) were administered a standard diet containing 10% kcal from fat. Mice in the HF group (n=7) were fed with a home-made high-fat diet that contained 58% kcal from fat, 27% kcal from carbohydrate and 15% kcal from protein (12) for 12 weeks. The remaining mice were fed with a high-fat diet for eight weeks and then supplemented with the PPARγ agonist RSG (Sigma-Aldrich, St. Louis, MO, USA) at a dose of 10 mg/kg high-fat diet for an additional four weeks as the RSG group (n=7). The dose of RSG was selected based on a previously published study (13). RSG was mixed with the powder diet. Food and water were available *ad libitum*. All the mice were maintained with a control temperature of 20±2°C, a relative humidity of 50±10% and a 12-h light/dark cycle. Animal experimental procedures were conducted under an animal protocol approved by the Institutional Animal Care and Use Committee of China Medical University (Shenyang, Liaoning, China).

### Serum biochemical and hormonal measurements

Blood samples were collected from the abdominal aorta. Serum glucose concentration was measured by the glucose oxidase method. Serum triglycerides and total cholesterol were determined using commercial kits (Sigma-Aldrich, St. Louis, MO, USA). The levels of serum adiponectin, leptin and insulin were determined by the ELISA kit (Boster Biotechnology, Wuhan, Hubei, China).

### Adipose tissue samples

At the end of the 12 weeks, all the mice were sacrificed. The weights of the inguinal and epididymal fat pad samples were determined immediately, and subsequently stored at −80°C for reverse transcription quantitative polymerase chain reaction (RT-qPCR) and western blot analysis.

### RT-qPCR analysis

The total RNA of the subcutaneous and epididymal adipose tissues was extracted using RNAiso plus (Takara, Dalian, Liaoning, China) according to the manufacturer’s instructions. The reverse transcription and PCR reactions were performed using the ABI Prism Real-time PCR 7500 system (Applied Biosystems, Inc., Foster City, CA, USA) as described previously (14). *β-actin* was used as the endogenous control gene. RT-qPCR data were analyzed using the 2^−ΔΔCT^ method (15). The sequences of the forward and reverse primers are listed in Table I.

### Western blot analysis

Briefly, epididymal and inguinal fat pads were homogenized in lysis buffer (50 mM Tris-HCl, pH 7.5, 0.1 mM Na_3_VO_4_, 1% Nonidet P-40, 25 mM NaF, 2 mM EDTA, 2 mM EGTA, 1 mM DTT and 1% protease inhibitor mixture; Sigma-Aldrich, St. Louis, MO, USA). Protein samples were boiled for 5 min in 1 × SDS sample buffer (50 mM Tris-HCl, pH 6.8, 20% glycerol, 2% SDS and 0.02% bromophenol blue) containing 2%-mercaptoethanol. The proteins on the gels that were separated by SDS-PAGE were transferred onto a polyvinylidene difluoride membrane for 3 h at 4°C. The membrane was blocked with 5% skimmed milk for 1 h at room temperature and incubated with goat anti-mouse *Gpc4* polyclonal antibodies (1:300 dilution; Santa Cruz Biotechnology, Santa Cruz, CA, USA) overnight at 4 °C, followed by horseradish peroxidase-conjugated secondary antibodies (1:5000 dilution; Pierce, Rockford, IL, USA) for 45 min at room temperature. Enhanced chemiluminescence reagent (Amersham Biosciences, Buckinghamshire, UK) was used to obtain signals. The blots were quantified using Scion Image 4.0 software (16).

### Statistical analysis

All the data were expressed as the mean ± standard deviation. Statistical analysis was conducted by the Student’s *t*-test within the same group and one-way analysis of variance among different groups using SPSS 13.0 software (SPSS, Inc., Chicago, IL, USA). P<0.05 was considered to indicate a statistically significant difference.

## Results

### Body weight, energy balance and fat distribution

After 12 weeks of feeding, mice in the RSG and HF groups had a significantly higher body weight, body weight gain, food efficiency, subcutaneous fat and epididymal mass than that of mice in CON group. RSG treatment had no influence on the amount of food intake (4.0±0.3 g/d in the CON group vs. 4.1±0.3 g/d in the HF group vs. 4.1±0.3 g/d in the RSG group, P>0.05). After 4 weeks of RSG treatment, mice in the RSG group showed a significant increase in subcutaneous fat weight compared with the mice in the HF group (0.8±0.2 g in the RSG group vs. 0.3±0.1 g in the HF group, P<0.05). However, there was no difference in the epididymal fat weight between the RSG-treated and untreated HF mice (Table II).

### Plasma glucose, serum hormones and lipids

As shown in Table III, plasma fasting glucose had significantly decreased in the RSG-treated mice compared with the untreated HF mice (P<0.05). Fasting insulin in the RSG-treated HF mice maintained the same concentration as that in the CON mice, but was markedly lower than that in the untreated HF mice (P<0.05). Serum leptin had significantly increased in the RSG-treated mice compared with the CON mice (P<0.05), but significantly decreased compared with the untreated HF mice (P<0.05). Serum adiponectin in the RSG-treated mice was higher than that in the untreated HF mice (P<0.05). The concentration of serum triglyceride had significantly decreased in the RSG-treated mice compared with the untreated HF mice (P<0.05).

### Expression of Gpc4 mRNA and protein in subcutaneous and visceral fat

**Figure 1:**
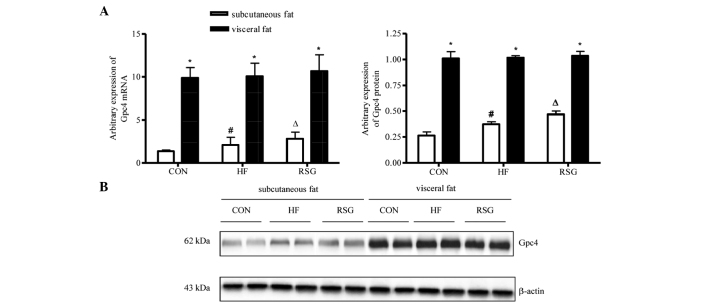
Comparison of the expression of *Gpc4* mRNA and protein in subcutaneous and visceral fat in standard diet, HF and RSG-treated C57BL/6J mice. Expression levels of Gpc4 (A) mRNA and (B) protein were measured by quantitative reverse transcription polymerase chain reaction and western blot analysis. Differences in the same depot from the three groups were analyzed by analysis of variance, and differences in the subcutaneous (open columns) and visceral fat (filled columns) within the same group were analyzed by the Student’s t-test. All the data are expressed as the mean ± standard deviation. ^*^P<0.05 vs. subcutaneous fat; ^#^P<0.05 vs. CON; ^Δ^P<0.05 vs. HF. Gpc4, glypican 4; CON, control, HF, high-fat feeding; RSG, rosiglitazone.

As shown in Fig. 1A and B, the expression of *Gpc4* mRNA and protein was significantly higher in visceral than in subcutaneous fat in all three groups (P<0.05). The mice in the HF group had increased *Gpc4* mRNA and protein expression levels in subcutaneous fat compared with mice in the CON group. After 4 weeks of RSG treatment the expression of *Gpc4* mRNA and protein in subcutaneous fat increased significantly compared with that of the untreated HF mice (P<0.05). However, no significant difference was detected for the expression of *Gpc4* mRNA and protein in visceral fat among all three groups.

### Expression of Sp1 and Sp3 mRNA and Sp3/Sp1 ratio in subcutaneous and visceral fat

**Figure 2:**
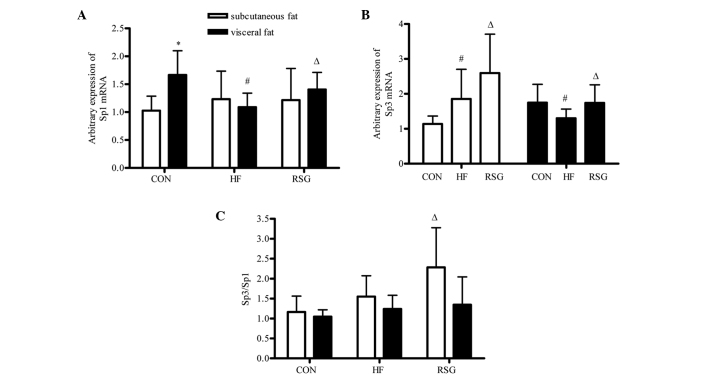
Comparison of *Sp1* and *Sp3* mRNA expression and *Sp3/Sp1* ratio in subcutaneous and visceral fat in standard diet, HF and RSG-treated C57BL/6J mice. (A) Expression of *Sp1* mRNA, (B) expression of *Sp3* mRNA and (C) the *Sp3*/*Sp1* ratio in subcutaneous and visceral fat in the CON, HF and RSG groups. The *Sp3*/*Sp1* ratio was obtained by the arbitrary expression of *Sp3* mRNA/the arbitrary expression of *Sp1* mRNA. All the data are expressed as the mean ± standard deviation. ^*^P<0.05 vs. subcutaneous fat; ^#^P<0.05 vs. CON; ^Δ^P<0.05 vs. HF. CON, control, HF, high-fat feeding; RSG, rosiglitazone; Sp, specificity protein.

As shown in Fig. 2A, the expression of *Sp1* mRNA in visceral fat was significantly higher than that in subcutaneous fat within the CON group (P<0.05), but no difference was detected in the HF and RSG groups. Compared with the CON and RSG groups the expression of *Sp1* mRNA in visceral fat was markedly decreased in the HF group (P<0.05). As shown in Fig. 2B, the expression of *Sp3* mRNA in subcutaneous fat in the HF group was significantly higher than that in the CON group, but lower than that in the RSG group (P<0.05). The *Sp3*/*Sp1* ratio in subcutaneous fat in the RSG group was significantly higher than that in the CON and HF groups (P<0.05), as shown in Fig. 2C.

## Discussion

RSG, an agonist of PPARγ, exerts multifunction via the stimulation of PPARγ on a number of cell types (17,18) and is used clinically to treat type 2 diabetes mellitus. In the present study, a section of the results obtained were similar to previous studies (19,20). RSG decreased serum glucose and insulin, decreased the synthesis of leptin and triglyceride, and increased the synthesis of adiponectin (21). The results also showed that RSG promoted subcutaneous fat gain and had little effect on visceral fat accumulation, which implied that PPARγ activation in subcutaneous fat tissues exerts a higher ability to increase fat accumulation than that in visceral fat tissues in mice feeding with a high-fat diet. The possible mechanisms leading to the effects of RSG treatment include the differentiation of preadipocytes from subcutaneous regions being markedly augmented compared with that from visceral regions (8). However, another study showed that there was no difference in preadipocytes between subcutaneous and visceral sites and they were enhanced to the same extent by treating with RSG in the two depots (22). The second possible mechanism is that RSG treatment may be associated with the depot-specific regulation of lipid uptake, storage and energy expenditure genes in visceral and subcutaneous fat (9–11). These studies demonstrated that RSG treatment resulted in the increase in the subcutaneous fat accumulation and reduction in visceral fat accretion by stimulating lipid uptake in subcutaneous fat and increasing energy expenditure greatly in visceral fat. Another hypothesized mechanism is that certain developmental genes are differentially expressed in adipocytes derived from subcutaneous and visceral depots, which may play an important role in body fat distribution. *Gpc4* is a gene known to exert a role in early development and pattern specification. However, a recent study identified *Gpc4* as a novel adipokine capable of enhancing insulin signaling and modulating adipocyte differentiation (23), indicating a potentially important role of body fat regulation. Gesta *et al* (5) demonstrated that different adipocyte precursors are responsible for a specific fat depot development and may be involved later in the functional differences observed between visceral and subcutaneous adipose depots. The present study showed that in all groups the expression of *Gpc4* mRNA and protein was significantly higher in visceral than in subcutaneous fat, which was similar to a previous study that indicated a difference in *Gpc4* expression in subcutaneous and visceral adipose tissues (5). The mice treated with RSG were also found to have increased *Gpc4* mRNA and protein expression levels in subcutaneous fat compared with untreated HF mice, but no difference was measured in the visceral depot between HF and RSG-treated mice. Thus, the present results indicated that the levels of *Gpc4* mRNA and protein expression in subcutaneous adipose tissue were associated with PPARγ activation, and may play an important role in fat distribution. Indeed, the expression of *Gpc4* in subcutaneous fat has a strong correlation with BMI in humans, and high levels of *Gpc4* expression in visceral adipose tissues and low levels in subcutaneous adipose tissues appear to be linked with high WHR (5). In addition, circulating Gpc4 levels had a significant positive correlation with the WHR and the ratio of visceral to subcutaneous fat area, indicating the association of Gpc4 with body fat distribution and insulin resistance (24).

Sp1 and Sp3 have been reported as major activators of the *Gpc4* promoter. They play a significant role in the regulation of the *Gpc4* gene expression (4). The levels of *Gpc4* expression changed with the Sp3 protein and the ratio of Sp3 to Sp1 in the process of differentiation of 3T3-F442A cells from adipoblasts to differentiated adipocytes. Therefore, it was hypothesized that Sp1 and Sp3 are perhaps involved in regulating *Gpc4* expression in the process of PPARγ activation in subcutaneous and visceral fat tissues. The results showed that the expression of *Sp3* mRNA, the ratio of *Sp3*/*Sp1* in subcutaneous fat and the expression of *Sp3* and *Sp1* mRNA in visceral fat in RSG-treated mice had significantly increased compared with the mice in the HF group, while no statistical difference was observed in the expression of *Sp1* mRNA in the subcutaneous fat in the HF and RSG groups. The results indicated that PPARγ activation affected the expression of *Sp1* and *Sp3* either in subcutaneous and/or visceral fat. A possible explanation included that the activation of PPARγ induces an unknown gene that is involved in the Sp1 or Sp3 transcriptional activity. In addition, it is possible that PPARγ directly regulated the expression of a corepressor that interacts with Sp1 or Sp3. Several studies have indicated that there is a physical interaction between Sp1 and PPARγ (25,26). Activation of PPARγ decreased Sp1 activity by modulation of glycosylation and the GlcNAcylation status of Sp1, but no direct interaction was detected between Sp1 and PPARγ (27,28). Notably, in the present study, the ratio of *Sp3* to *Sp1* was consistent with the expression of *Gpc4* not only in subcutaneous fat but also in visceral fat indicating that the ratio of Sp3 to Sp1 perhaps regulated *Gpc4* expression in the process of PPARγ activation. However, the direct effect among PPARγ, Sp3, Sp1 and Gpc4 expression was not detected, therefore further study is required to resolve how the activation of PPARγ affects the expression of Sp1 and Sp3, and how the change of Sp1/Sp3 affects the expression of the *Gpc4* gene.

In conclusion, PPARγ activation was demonstrated to increase the *Gpc4* mRNA and protein expression in subcutaneous adipose tissues but had no effect on visceral adipose tissues, which was consistent with the change of the ratio of *Sp3/Sp1* in these depots. The present study indicated that *Gpc4* may play an important role in fat distribution, and this effect is possibly regulated by the ratio of *Sp3/Sp1* in the subcutaneous and visceral fat tissues.

## Figures and Tables

**Table I:** Oligonucleotide sequences and product sizes of polymerase chain reaction primers.

Gene	Sense (5′-3′)	Antisense (5′-3′)	Size, bp
*β-actin*	CATCCGTAAAGACCTCTATGCCAAC	ATGGAGCCACCGATCCACA	171
Glypican 4	AGAGCAACGCCCAACCAC	GCCATTCCAGCAGTCATC	169
*Sp1*	GGCCTCCAGACCATTAACCTCA	TCATGTATCCCATCACCACCAGA	149
*Sp3*	AGATGATGCCTTGATTACTG	ATGTCTTGATTGCTGGTG	114

Sp, specificity protein; bp, base pairs.

**Table II:** Data of body weight, energy balance and fat distribution in standard diet, HF and RSG-treated C57BL/6J mice.

Variable	CON (n=7)	HF (n=7)	RSG (n=7)
Initial body weight, g	19.9±1.0	20.2±0.6	20.5±0.7
Final body weight, g	27.9±1.8	31.2±2.9^a^	32.5±1.9^a^
Body weight gain, g	7.7±1.0	11.1±2.7^a^	11.9±1.6^a^
Food intake, g	4.0±0.3	4.1±0.3	4.1±0.3
Food efficiency, %^b^	192.5	268.3^a^	290.2^a^
Subcutaneous fat, g	0.07±0.02	0.3±0.1^a^	0.8±0.2^a^,^c^
Visceral fat, g	0.4±0.08	1.2±0.4^a^	1.1±0.4^a^

Differences among the three groups were analyzed by analysis of variance. Data are presented as the mean ± standard deviation of seven mice.

a P<0.05 vs. CON;

b calculated as g body weight gain/100 g food ingested;

c P<0.05 vs. HF.

CON, control; HF, high-fat feeding; RSG, rosiglitazone.

**Table III:** Serum biochemical and hormonal data in standard diet, HF and RSG-treated C57BL/6J mice.

Variable	CON (n=7)	HF (n=7)	RSG (n=7)
Fasting glucose, mmol/l	0.4±0.06	2.0±0.5^a^	0.6±0.2^b^
Fasting insulin, ng/ml	0.2±0.04	0.4±0.1^a^	0.2±0.05^b^
Leptin, ng/ml	10.7±2.7	33.3±3.7^a^	25.5±2.9^a^,^b^
Adiponectin, ng/ml	24.5±3.1	20.1±2.0^a^	32.1±4.8^a^,^b^
Triglyceride, mmol/l	1.6±0.1	1.9±0.4^a^	1.6±0.3^b^
Cholesterol, mmol/l	2.3±0.3	3.4±0.7^c^	3.8±0.6^c^

All data are expressed as the mean ± standard deviation and analyzed by analysis of variance.

a P<0.05 vs. CON,

b P<0.05 vs. HF;

c P<0.01 vs. CON.

CON, control; HF, high-fat feeding; RSG, rosiglitazone.
